# Fighting the Dengue Virus

**DOI:** 10.7759/cureus.1271

**Published:** 2017-05-24

**Authors:** Aniqa Faraz, Muhammad Aadil, Muhammad Nabeel Shafqat

**Affiliations:** 1 Department of Internal Medicine, King Edward Medical University Lahore, Pakistan; 2 Department of Psychiatry and Mental Health, Rush University Medical Center; 3 Department of Internal Medicine, University of Medical Sciences "Serafin Ruiz De Zarate" Villa Clara (Ucmvc)

**Keywords:** dengue virus, dengue vaccine

## Abstract

The incidence of dengue has been on the upsurge in the last decade. It has affected around one-third of the world's population living in endemic areas. It can be asymptomatic or may present with some specific symptoms. No control measures have proven beneficial to decrease the prevalence of this disease. The emergence of dengue vaccine has been a revolutionary hope in the future of patients affected with this disease. No doubt, this vaccine has its limitations and may do more harm than good, but with correct use, it can prove to be the most beneficial step taken in managing dengue so far.

## Editorial

Dengue is a mosquito-borne viral illness transmitted to humans by Aedes aegypti. This mosquito is most commonly found in the tropical and subtropical regions of the world. The global incidence of this virus has increased dramatically in the last decades placing more than one-third of the world population living in endemic areas at risk, with an annual incidence of 400 million cases [1].

There has been no evidence of a decline in the spread of dengue with dengue prevention and control programs as the endemic countries have limited resources and control measures are brought into effect only in epidemics. So the arrival of dengue virus vaccine is indispensable to prevent complications and death from dengue fever [2].

Four distinct serotypes have been documented for causing dengue: dengue virus one (DENV-1), dengue virus two (DENV-2), dengue virus three (DENV-3), and dengue virus four (DENV-4). Infection by one serotype confers a life-long immunity to that virus, but a subsequent infection with another serotype causes a more severe dengue disease with a mortality rate of 1-20% [2]. The patients infected with dengue virus may be asymptomatic or may present with a severe illness sometimes leading to death.

Symptomatic individuals may present with fever, rash, headache, pain behind the eyes, muscle aches, bone and joint pains, nausea, and vomiting [1]. The atypical manifestations of dengue include encephalopathy, hepatitis, hemolytic-uremic syndrome, myocarditis, acute respiratory distress syndrome, and myositis. The most common complications include hemorrhage and shock [3].

An effective dengue vaccine is an important strategy to effectively control the disease burden. Sanofi Pasteur vaccine is the only one that made it through phase III trials. It is a tetravalent chimeric vaccine with marked efficacy against serotype three and four. It is also known to be the safest one. According to the World Health Organization, in 2016 it was approved for ages 9-45 years (Brazil) and 9-60 years (Paraguay); three doses to be given six months apart [4-5].

Dengvaxia, the dengue vaccine, is formed by splicing four dengue serotypes with the yellow fever virus 17D genes. With phase III trial under investigation, after two years of this trial, it was proven that Dengvaxia has better efficacy in those already immune to one of the serotypes, but has less efficacy in those with no prior infection, questioning the usefulness of this vaccine in travelers to endemic areas. However, this vaccine significantly reduced the prevalence of mild and severe dengue and the rate of hospitalization in those previously affected. According to the Study on Global Ageing and Adult Health run by the World Health Organization, the greatest benefit is achieved in seropositive patients at the time of vaccination.

### Expert opinion

The goal of eradication of the epidemic of dengue can only be achieved by providing access to the low-cost vaccine in an abundant supply to endemic countries and implementing the appropriate measures of control like an effective water supply system, waste disposal, and the use of protective clothing and insecticide spray in travelers to these regions.

No doubt, dengue vaccine is a new hope to combat the illness and hospitalization caused by dengue, but its use is limited to places where it will do more good than harm.

There is still uncertainty about this vaccine and more research needs to be done on the dose and the selection of recipients for this vaccine. Its effectiveness depends on a number of factors including the age of the patient receiving the vaccine, the type of dengue, previous infection with dengue, and the local epidemiology.

The vaccine will win the battle if serological tests are done before the use of the vaccine. The correct use of this vaccine in future will decrease the number of severe cases and hospitalizations leading to a significant achievement.
